# Heart failure and major haemorrhage in people with atrial fibrillation

**DOI:** 10.1136/openhrt-2024-002975

**Published:** 2024-10-14

**Authors:** Nicholas R Jones, Margaret Smith, Sarah Lay-Flurrie, Yaling Yang, FD Richard Hobbs, Clare J Taylor

**Affiliations:** 1 Nuffield Department of Primary Care Health Sciences, University of Oxford, Oxford, UK; 2 NIHR Oxford Biomedical Research Centre, Oxford, UK; 3 Department of Applied Health Sciences, University of Birmingham, Birmingham, UK

**Keywords:** Atrial Fibrillation, HEART FAILURE, ANTICOAGULATION, EPIDEMIOLOGY

## Abstract

**Background:**

Heart failure (HF) is not included in atrial fibrillation (AF) bleeding risk prediction scores, reflecting uncertainty regarding its importance as a risk factor for major haemorrhage. We aimed to report the relative risk of first major haemorrhage in people with HF and AF compared with people with AF without HF (‘AF only’).

**Methods:**

English primary care cohort study of 2 178 162 people aged ≥45 years in the Clinical Practice Research Datalink from January 2000 to December 2018, linked to secondary care and mortality databases. We used traditional survival analysis and competing risks methods, accounting for all-cause mortality and anticoagulation.

**Results:**

Over 7.56 years median follow-up, 60 270 people were diagnosed with HF and AF of whom 4996 (8.3%) had a major haemorrhage and 36 170 died (60.0%), compared with 8256 (6.4%) and 34 375 (27.2%), respectively, among 126 251 people with AF only. Less than half those with AF were prescribed an anticoagulant (45.6% from 2014 onwards), although 75.7% were prescribed an antiplatelet or anticoagulant. In a fully adjusted Cox model, the HR for major haemorrhage was higher among people with HF and AF (2.52, 95% CI 2.44 to 2.61) than AF only (1.87, 95% CI 1.82 to 1.92), even in a subgroup analysis of people prescribed anticoagulation. However, in a Fine and Gray competing risk model, the HR of major haemorrhage was similar for people with AF only (1.82, 95% CI 1.77 to 1.87) or HF and AF (1.71, 95% CI 1.66 to 1.78).

**Conclusions:**

People with HF and AF are at increased risk of major haemorrhage compared with those with AF only and current prediction scores may underestimate the risk of haemorrhage in HF and AF. However, people with HF and AF are more likely to die than have a major haemorrhage and therefore an individual’s expected prognosis should be carefully considered when predicting future bleeding risk.

**WHAT IS ALREADY KNOWN ON THIS TOPIC**

- People with both heart failure and atrial fibrillation are at high risk of stroke and should be considered for anticoagulation. However, people with heart failure are not identified as being at high risk of bleeding based on current risk prediction scores.

**WHAT THIS STUDY ADDS**

- Heart failure was associated with an increased relative risk of major haemorrhage among people with atrial fibrillation including among those treated with anticoagulation, though when assessing risk, it is important to consider the poor prognosis for some people with heart failure.

**HOW THIS STUDY MIGHT AFFECT RESEARCH, PRACTICE OR POLICY**

- Clinicians should consider people with heart failure to be at increased risk of bleeding when starting anticoagulation and seek to address any modifiable bleeding risk factors. Future research could seek to update bleeding risk prediction scores to account for competing risks and review the need to include heart failure as a risk factor.

## Introduction

 Heart failure (HF) and atrial fibrillation (AF) commonly coexist due to shared risk factors, and the presence of either condition predisposes an individual to developing the other.^1^ A recent population cohort study reported the lifetime risk of developing HF among people with AF is 41.2% (95% CI 39.8% to 42.7%).^2^ HF is included as a risk factor for stroke in CHA_2_DS_2_-VASc, meaning everybody with AF and HF should be considered for anticoagulation as stroke prevention.^3 4^

However, anticoagulants are also associated with an increased risk of haemorrhage,^5^ and bleeding related to anticoagulants is a leading cause of hospital admissions due to adverse drug reactions.^6 7^ Clinicians should, therefore, assess a patient’s risk of major haemorrhage when considering anticoagulation using a validated risk prediction score, such as HAS-BLED (Hypertension, Abnormal renal or liver function, Stroke, Bleeding history or predisposition, Labile INR, Elderly, Drugs or alcohol concomitantly) or ORBIT (derived from the Outcomes Registry for Better Informed Treatment of Atrial-Fibrillation).^8 9^ Particular attention should be given to addressing bleeding risk factors among high-risk patients. Neither HAS-BLED nor ORBIT include HF as a predictor, so this does not currently inform the risk assessment.^8 9^ This reflects uncertainty as to the extent to which HF is associated with a risk of bleeding and whether it is a useful predictor of major haemorrhage in people with AF.

People with HF typically have a poor prognosis with around 50% dying within 5 years of diagnosis.^10^ When estimating the incidence of bleeding, it is important to consider this ‘competing risk’ of death, because those who die are no longer at risk of the primary outcome.^11^ Traditional analytical methods that do not account for competing risks tend to overestimate the prognostic significance of a variable,^11^ particularly in conditions such as HF where the mortality rate is high.

In this study, we aim to determine the relative risk of major haemorrhage in people with both HF and AF, compared with people with AF only, among a large, primary care cohort and accounting for the competing risk of death.

## Methods

We conducted a retrospective cohort study using routinely collected primary care data in England within Clinical Practice Research Datalink (CPRD) GOLD between 1 January 2000 and 31 December 2018. Records were linked to inpatient secondary care data from Hospital Episode Statistics (HES) and to the Office for National Statistics (ONS) for mortality data. An Independent Scientific Advisory Committee approved access to CPRD data (reference 19_125).

### Patient and public involvement

A group of 10 people with lived experience of HF and other cardiovascular diseases discussed the plan for this analysis at inception, as part of a wider project looking at stroke and mortality in people with HF and AF. The group confirmed the importance of this clinical topic for patients and their carers. They called for urgent action not only to help implement local and national guidelines to improve anticoagulation prescribing but also to improve risk scores for stroke and bleeding if possible, with a view to minimising harm from treatment.

### Study population

From CPRD GOLD, we included people aged ≥45 years, who were eligible for data linkage and registered at an ‘up-to-standard’ practice that had contributed data to CPRD for a minimum of 1 year.^12^ People entered the cohort on the latest of the following dates, defined as their cohort entry date: 1 January 2000, 45th birthday date, date of registration at a participating practice plus 1 year or date a practice was registered as up-to-standard plus 1 year. People left the cohort on the earliest of the following dates: 31 December 2018, death, end of practice data collection, last available linked data or transfer out of CPRD registered practice. We excluded people who had a history of major haemorrhage prior to their cohort entry date, as all people with a previous major haemorrhage are likely to be identified as being at high risk of a recurrent event. This study was part of a larger analysis of stroke incidence and people with a stroke prior to their cohort entry date were also excluded, but not those with a stroke during follow-up.

### Exposure

People were categorised as having AF without HF (‘AF only’) or HF without AF (‘HF only’) based on the first diagnostic code for either condition in either the primary or secondary care record. People were categorised as having both HF and AF from the first date the other condition was coded in either the primary or secondary care record. This included all eligible patients with an existing diagnosis of HF or AF prior to their cohort entry date and incident diagnoses during follow-up. For time-to-event analyses, we excluded people diagnosed with HF or AF prior to their cohort entry date. HF and AF were treated as time-varying covariates, meaning that people could move between these exposure groups across follow-up.

### Outcomes

The primary outcome was first major haemorrhage, as recorded in either CPRD or HES. We defined major haemorrhage as an event leading to hospital admission or death, or bleeding in a critical organ (intracranial, intra-articular, massive haemoptysis and upper gastrointestinal), in line with previous observational research.^13 14^ Secondary outcomes included subcategories of haemorrhage, haemorrhage-related mortality, and incidence of major haemorrhage by age and in relation to anticoagulation or antiplatelet treatment.

### Covariates

All covariates were defined by the most recent relevant code in a patient’s primary care record prior to their cohort entry date or before a diagnosis of HF or AF. We included all elements of the CHA_2_DS_2_-VASc score, except for HF and the variables in the HAS-BLED and ORBIT bleed risk scores, excluding major haemorrhage. Data were collected on smoking status, ethnicity, body mass index, frailty, migraine and total number of prescribed medications and significant comorbidities. Anticoagulation and antiplatelets were identified by a prescription within the first 90 days following their cohort entry date or date of diagnosis with HF or AF.

### Statistical analysis

We used descriptive statistics to compare demographic information and bleed risk scores for the study population at their cohort entry date based on the presence or absence of HF and AF. We report both crude and age-stratified (<65, 65 to 74 or ≥75 years) incidence rates of major haemorrhage per 1000 person years at risk by exposure group.^15 16^

A Cox-proportional hazards model was used for the primary analysis, estimating the relative risk of a first major haemorrhage in people with HF and AF, compared with people with AF only, HF only or neither condition. We chose to compare the relative risk of major haemorrhage against the general population to provide an insight into the relative magnitude of risk for people with AF or HF and AF.

We conducted a Fine and Gray competing risk analysis to estimate the subdistribution HR for risk of first major haemorrhage, where all-cause mortality (excluding death related to a major haemorrhage) was defined as the competing risk. A competing risk methods summary paper by Lau *et al* outlines that cause-specific hazard models, such as the Cox model, ‘may be better suited for studying the aetiology of diseases, whereas the subdistribution hazard model (eg, Fine and Gray) has used in predicting an individual’s risk’.^17^

Both the Cox and Fine and Gray models were first adjusted for age and sex, then for the stroke risk factors in CHA_2_DS_2_-VASc, plus smoking status and ethnicity and finally adjusted for the bleeding risk factors in HAS-BLED and ORBIT, which were included as time-varying covariates. The cumulative incidence function (CIF) was estimated from the Fine and Gray model and compared with the Kaplan-Meier (KM) failure function curve. For the CIF and KM curves, people were censored when their HF or AF status changes, including either the date they developed AF or HF, or the date they were first diagnosed with both conditions.

We conducted a landmark analysis including all cases where the cohort entry date was within the year 2000 and repeated this for each year up to 15-year follow-up. For each calendar year, we used the *sts list* and *stcomlist* packages to give an estimate of the unadjusted KM failure function and CIF for major haemorrhage at 3 months, 1, 2, 5 and 10-year follow-up.^11 18^ We report the mean of these results weighted using the inverse of the variance. The KM failure curve for each landmark year was plotted to check for consistency of results across the landmark years. The CIF provides an estimate of the marginal probability for a cause-specific event, that is, the probability a patient would have a major haemorrhage, but accounting for the fact that once an individual dies, they are no longer at risk of haemorrhage.^11^

In a subgroup analysis, we report the relative risk of major haemorrhage among people with AF who were prescribed an anticoagulant, given this is the population for whom previous bleeding risk prediction scores are intended. We also conducted a postestimation analysis of differences in HR for major haemorrhage based on exposure to anticoagulation and antiplatelet medication and compared risk of major haemorrhage by age bands.

In a sensitivity analysis, we repeated the primary analysis using a wider definition of haemorrhage, including other common types of bleed (online supplemental appendix 1). A second sensitivity analysis included deaths related to major bleeding that were reported in the ONS death registry, but not in CPRD or HES. Finally, the validity of using competing risks models with time-varying covariates has been questioned,^19^ so we conducted a landmark analysis from each person’s cohort entry date and 5-year follow-up including HF and AF as time-independent covariates.^20^

There were substantial missing data for smoking status and ethnicity, which were felt to be unlikely to be missing completely at random. We not only created a ‘missing’ variable category for each in the main analysis but also conducted a complete case analysis for comparison.

The analysis was completed using Stata V.14 (StataCorp, Texas).

## Results

We included 2 178 162 people (mean age 56.6 years, 51.9% women), of whom 60 270 had both HF and AF, 79 461 had HF only and 126 251 had AF only at some point during the study period (table 1). The median follow-up time was 7.56 years (range 0–18.9 years). Among people diagnosed with both HF and AF during follow-up, 15 419 were diagnosed with HF first, 30 708 were diagnosed with AF first and 10 190 people diagnosed with HF and AF on the same day. The median time to a subsequent diagnosis of AF among people with pre-existing HF was 1.69 years (IQR 0.36–4.53 years) and the median time to a diagnosis of HF among people with pre-existing AF was 1.95 years (IQR 0.40 to 5.08 years).

**Table 1:** Baseline table, showing characteristics based on the presence of heart failure and/or atrial fibrillation at cohort entry date

	Neither atrial fibrillation nor heart failure	Heart failure only	Atrial fibrillation only	Atrial fibrillation and heart failure	Total
Number of participants	2 105 805	25 588	33 905	12 864	2 178 162
Age at entry into study (years), mean (SD)	56.0 (12.3)	75.9 (12.3)	72.4 (13.0)	78.3 (11.0)	56.6 (12.8)
Patient sex					
Female	1 093 306 (51.9%)	14 301 (55.9%)	16 268 (48.0%)	7072 (55.0%)	1 130 947 (51.9%)
Male	1 012 499 (48.1%)	11 287 (44.1%)	17 637 (52.0%)	5792 (45.0%)	1 047 215 (48.1%)
Ethnicity					
White	1 460 396 (69.4%)	20 979 (82.0%)	29 951 (88.3%)	11 232 (87.3%)	1 522 558 (69.9%)
Other ethnicity*	90 876 (4.3%)	1083 (4.2%)	808 (2.4%)	298 (2.3%)	93 065 (4.3%)
Unknown	554 533 (26.3%)	3526 (13.8%)	3146 (9.3%)	1334 (10.4%)	562 539 (25.8%)
Smoking status					
Non-smoker	977 286 (46.4%)	10 979 (42.9%)	16 293 (48.1%)	5875 (45.7%)	1 010 433 (46.4%)
Current smoker	424 797 (20.2%)	3420 (13.4%)	3695 (10.9%)	1218 (9.5%)	433 130 (19.9%)
Ex-smoker	405 107 (19.2%)	7189 (28.1%)	9718 (28.7%)	3849 (29.9%)	425 863 (19.6%)
Missing	298 615 (14.2%)	4000 (15.6%)	4199 (12.4%)	1922 (14.9%)	308 736 (14.2%)
Units of alcohol per week (mean (SD))	7.7 (11.8)	4.8 (9.5)	6.9 (11.9)	5.3 (10.4)	7.7 (11.8)
Body mass index (kg/m^2^, mean (SD))	26.6 (4.7)	27.7 (5.2)	26.8 (4.9)	27.0 (5.3)	26.6 (4.8)
Chronic kidney disease	18 314 (0.9%)	1488 (5.8%)	1809 (5.3%)	1386 (10.8%)	22 997 (1.1%)
Diabetes	99 744 (4.7%)	4528 (17.7%)	3681 (10.9%)	2278 (17.7%)	110 231 (5.1%)
Hypertension	381 505 (18.1%)	11 551 (45.1%)	15 018 (44.3%)	6107 (47.5%)	414 181 (19.0%)
Liver disease	7701 (0.4%)	109 (0.4%)	159 (0.5%)	50 (0.4%)	8019 (0.4%)
Migraine	112 257 (5.3%)	765 (3.0%)	1239 (3.7%)	342 (2.7%)	114 603 (5.3%)
Thrombo-embolism	11 279 (0.5%)	716 (2.8%)	800 (2.4%)	524 (4.1%)	13 319 (0.6%)
Vascular disease (including MI and PAD)	51 325 (2.4%)	7425 (29.0%)	3979 (11.7%)	2914 (22.7%)	65 643 (3.0%)
Frailty category					
Fit	1 977 141 (93.9%)	11 159 (43.6%)	20 309 (59.9%)	3865 (30.0%)	2 012 430 (92.4%)
Mild	119 997 (5.7%)	11 548 (45.1%)	11 656 (34.4%)	6566 (51.0%)	149 745 (6.9%)
Moderate	8274 (0.4%)	2626 (10.3%)	1765 (5.2%)	2154 (16.7%)	14 819 (0.7%)
Severe	393 (0.0%)	255 (1.0%)	175 (0.5%)	279 (2.2%)	1102 (0.1%)
Number of comorbidities					
0–1	1 559 806 (74.1%)	2494 (9.7%)	6696 (19.7%)	629 (4.9%)	1 569 625 (72.1%)
2–5	523 076 (24.8%)	17 889 (69.9%)	23 384 (69.0%)	8098 (63.0%)	572 447 (26.3%)
6–9	22 606 (1.1%)	4974 (19.4%)	3682 (10.9%)	3873 (30.1%)	35 135 (1.6%)
10+	317 (0.0%)	231 (0.9%)	143 (0.4%)	264 (2.1%)	955 (0.0%)
Number of prescribed medications					
0–1	1 139 623 (54.1%)	1328 (5.2%)	3597 (10.6%)	344 (2.7%)	1 144 892 (52.6%)
2–5	668 276 (31.7%)	6128 (23.9%)	11 964 (35.3%)	2353 (18.3%)	688 721 (31.6%)
6–9	208 104 (9.9%)	8894 (34.8%)	10 744 (31.7%)	4555 (35.4%)	232 297 (10.7%)
10+	89 802 (4.3%)	9238 (36.1%)	7600 (22.4%)	5612 (43.6%)	112 252 (5.2%)
Anticoagulant					
VKA	7197 (0.3%)	1222 (4.8%)	8579 (25.3%)	4562 (35.5%)	21 560 (1.0%)
DOAC	254 (0.0%)	19 (0.1%)	452 (1.3%)	202 (1.6%)	927 (0.0%)
Time in therapeutic range (%) for those on VKA (mean, SD)	47.9 (33.9)	54.3 (30.4)	64.3 (27.3)	60.9 (27.4)	56.7 (31.1)
Antiplatelet					
Standard dose	122 631 (5.8%)	11 031 (43.1%)	12 161 (35.9%)	5170 (40.2%)	150 993 (6.9%)
High dose	5278 (0.3%)	461 (1.8%)	755 (2.2%)	284 (2.2%)	6778 (0.3%)
Dual antiplatelet	2863 (0.1%)	494 (1.9%)	258 (0.8%)	156 (1.2%)	3771 (0.2%)
Other†	98 (0.0%)	1 (0.0%)	1 (0.0%)	0 (0.0%)	100 (0.0%)

Data are number (%), unless otherwise stated.

* Ethnicity was categorised into these groups given the relatively small numbers of patients in the cohort who had this information coded and were not white. Even among those who had a code for ethinicityethnicity that was not ‘White’, ‘Other’ ethnicity was most common (n=19 470), followed by Indian (n=17 979) and Black Caribbean (n=10 386).

† Other antiplatelets referred to combinations of an antiplatelet with another medication at a dose not typically used in cardiovascular treatment for example, aspirin 400mg / mg/ codeine 8mg mg tablets.

DOAC, direct oral anticoagulant; MI, myocardial infarction; PAD, peripheral arterial disease; VKA, vitamin K antagonist

People with HF and AF were more likely to be older than people with AF only (78.3 years vs 72.4 years) and women (55.0% vs 48.0%), with a greater prevalence of vascular disease (22.7% vs 11.7%), diabetes (17.7% vs 10.9%) and chronic kidney disease (10.8% vs 5.3%). They also had higher frailty scores, and a greater number of prescribed medications and co-morbid health conditions.

A greater proportion of people with HF and AF was categorised as being at high-risk of bleeding using either HAS-BLED (n=7061, 27.7%) or ORBIT (n=4466, 17.5%) compared with people with AF only (table 2).

**Table 2:** HAS-BLED and ORBIT bleeding risk scores among people with atrial fibrillation, with or without heart failure, at the time of atrial fibrillation diagnosis

	Number of patients	ORBIT score			HAS-BLED score		
		Low risk	Medium risk	High risk	Low risk	Medium risk	High risk
AF only	95 674	78 993 (82.6%)	9194 (9.6%)	7487 (7.8%)	39 204 (41.0%)	38 343 (40.1%)	18 127 (19.0%)
HF and AF	25 524	17 400 (68.2%)	3658 (14.3%)	4466 (17.5%)	7667 (30.0%)	10 796 (42.3%)	7061 (27.7%)
Total new AF patients	121 198	96 393 (79.5%)	12 852 (10.6%)	11 953 (9.9%)	46 871 (38.7%)	49 139 (40.5%)	25 188 (20.8%)

HAS-BLED is based on the following variables: Hypertension, Abnormal renal or liver function, Stroke, Bleeding history or predisposition, Labile INR, Elderly, Drugs or alcohol concomitantly. ORBIT is derived from the Outcomes Registry for Better Informed Treatment of Atrial-Fibrillation.

AFatrial fibrillationHFheart failure

### Incidence of major haemorrhage

**Figure 1:**
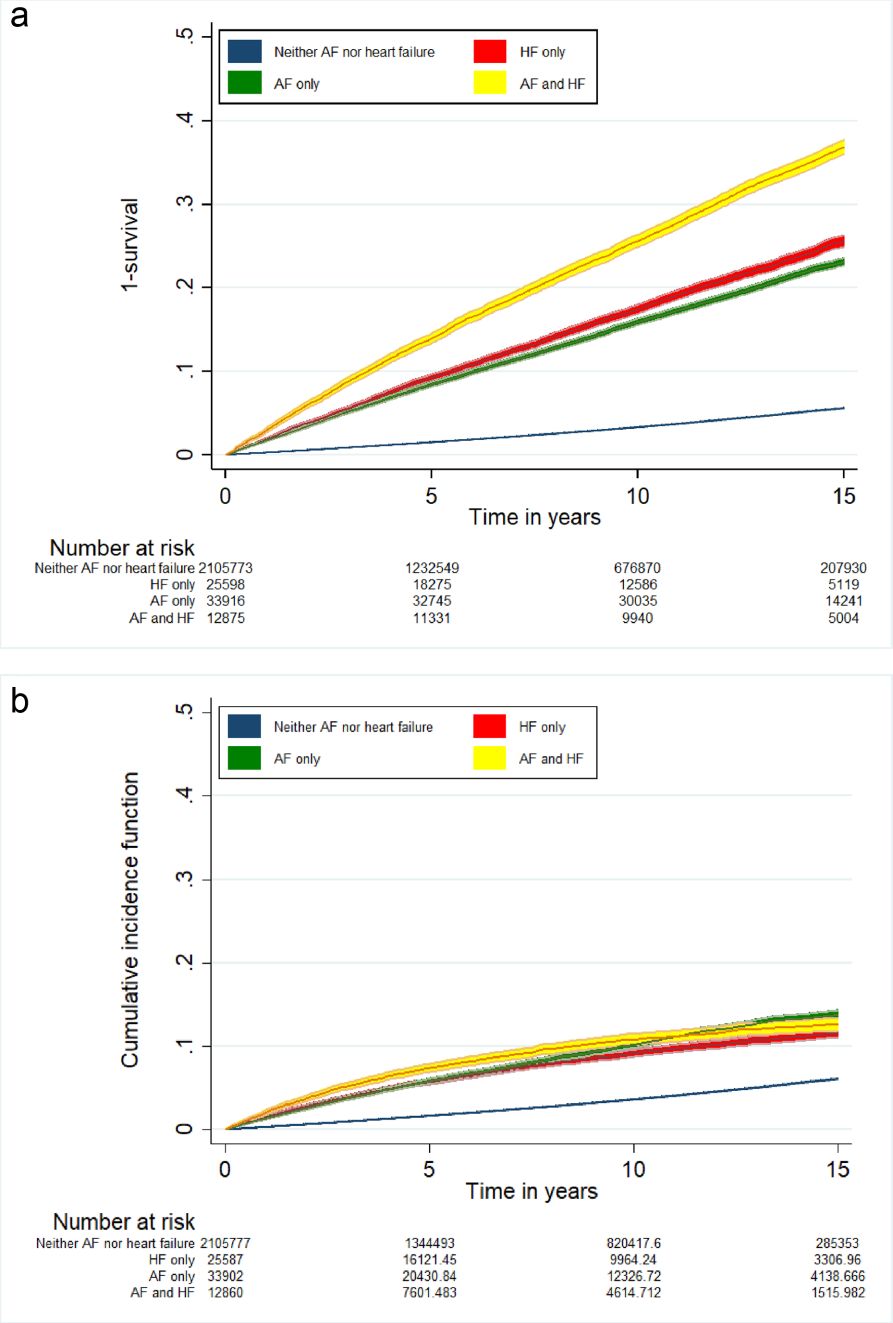
Comparison of the Kaplan-Meier failure function (1-survival) (Figure 1a) and cumulative incidence function (Figure 1b), showing the incidence of major haemorrhage over time between people with heart failure (HF) and/or atrial fibrillation (AF).

During the study, 72 196 (3.3%) people had a first major haemorrhage with a greater proportion among the group with HF and AF (n=4996, 8.3%) than AF only (n=8256, 6.4%) (table 3). Figure 1a shows the unadjusted cumulative probability of a first major haemorrhage is highest among people with HF and AF. The crude incidence rate (IR) in people with HF and AF (IR per 1000 person years at risk 30.7, 95% CI 29.9 to 31.6), was almost double that of AF only (IR 17.5, 95% CI 17.2 to 17.9) (online supplemental table 1).

**Table 3:** Proportion of participants to have a first major haemorrhage or death during follow-up, by type of haemorrhage and presence of heart failure and/or atrial fibrillation

	Heart failure and atrial fibrillation	Atrial fibrillation only	Heart failure only	Neither atrial fibrillation nor heart failure	Total
Number of participants at risk	60 270	126 251	79 461	2 105 773	2 178 162
Total number of deaths (% of population at risk)	36 170 (60.0%)	34 375 (27.2%)	34 691 (43.7%)	171 083 (8.1%)	276 319 (12.7%)
Number with a first haemorrhage (% of population at risk)	4995 (8.3%)	8256 (6.4%)	4709 (5.9%)	54 236 (2.6%)	72 196 (3.3%)
Proportion of major haemorrhages by type (% of total haemorrhages)					
Intracranial haemorrhage	895 (17.8%)	2069 (25.1%)	635 (13.5%)	10 410 (19.2%)	14 009 (19.5%)
Gastrointestinal haemorrhage	3481 (69.7%)	5326 (64.5%)	3598 (76.4%)	39 450 (72.7%)	51 855 (71.8%)
Other haemorrhage, of which:	619 (12.4%)	861 (10.4%)	476 (10.1%)	4376 (8.1%)	6332 (8.8%)
Haemoptysis	368	488	380	3681	4917
Haemoperitoneum	38	56	17	228	339
Haemarthrosis	86	153	32	308	579
Bleeding due to anticoagulant—hospitalised	127	164	47	159	497

Gastrointestinal bleeds were the most common category of major bleed (n=51 855), followed by intracranial haemorrhage (n=14 009) (table 3). The IR was higher for each category of major haemorrhage among people with HF and AF than people with AF only (online supplemental table 2).

### Relative risk of major haemorrhage

After adjusting for other bleeding risk factors, people with HF and AF were at greater relative risk of major haemorrhage (HR 2.52, 95% CI 2.44 to 2.61) compared with those with AF only (HR 1.87, 95% CI 1.82 to 1.92) (table 4).

**Table 4:** Hazard ratios for first major haemorrhage in people with atrial fibrillation and/or heart failure, comparing a Cox proportional hazards and a Fine and Gray competing risks model

	Number of participants at risk	Number of participants with a first major bleed	Unadjusted		Model 1		Model 2		Model 3	
			Cox model*	Fine and Gray model*	Cox model*	Fine and Gray model*	Cox model*	Fine and Gray model*	Cox model*	Fine and Gray model*
Neither heart failure nor atrial fibrillation	2 105 773	54 236	1 (ref)		1 (ref)		1 (ref)		1 (ref)	
Heart failure only	79 461	4709	5.37(5.21 to 5.53)	3.17(3.08 to 3.28)	2.36(2.29 to 2.43)	1.68(1.62 to 1.73)	2.10(2.03 to 2.16)	1.52(1.46 to 1.57)	1.98(1.92 to 2.05)	1.47(1.42 to 1.52)
Atrial fibrillation only	126 251	8256	4.67(4.56 to 4.78)	3.75(3.66 to 3.84)	2.18(2.12 to 2.23)	2.12(2.07 to 2.18)	1.98(1.93 to 2.03)	1.88(1.83 to 1.93)	1.87(1.82 to 1.92)	1.82(1.77 to 1.87)
Heart failure and atrial fibrillation	60 270	4995	8.20(7.97 to 8.45)	4.27(4.15 to 4.41)	3.12(3.02 to 3.21)	2.05(1.99 to 2.12)	2.72(2.63 to 2.80)	1.80(1.73 to 1.86)	2.52(2.44 to 2.61)	1.71(1.66 to 1.78)

Model 1 – —age and sex adjusted.

Model 2 – —adjusted for age, sex, hypertension, diabetes, history of previous stroke, thromboembolism or vascular disease, smoking and ethnicity.

Model 3 – —adjusted for age, sex, hypertension, chronic kidney disease, liver disease, previous stroke or thrombosis, non-steroidal anti-inflammatory or antiplatelet drugs, alcohol use and labile International Normalised Ratio for warfarin control.

* From the Cox model, we report the hazard ratioHR and from the Fine ineand Grayray model, we report the sub-distribution hazard ration. 95% confidence intervalsCI are shown in brackets.

In a sensitivity analysis including the broader definition of haemorrhage, 238 784 people had a first bleed, with haematuria (n=70 926) or rectal bleeding (n=60 509), the most frequent types. People with HF and AF were still at the greatest relative risk of haemorrhage (full model HR 2.11, 95% CI 2.06 to 2.16) (online supplemental table 2).

The sensitivity analysis including people who had a major haemorrhage recorded in ONS only produced similar results to the main analysis (online supplemental table 3).

### Major haemorrhage in the presence of competing risks

During the study, 276 319 (12.7%) people died. There were 36 170 (60.0%) deaths among those with HF and AF, 34 691 (43.7%) among people with HF only (43.7%, deaths) and 34 375 (27.2%) among those with AF only (table 3).

In the unadjusted Fine and Gray model, people with HF and AF remained at greater relative risk of major haemorrhage (HR 4.27, 95% CI 4.15 to 4.41) than people with AF only (HR 3.75, 95% CI 3.66 to 3.84) (table 4, figure 1b). However, in the fully adjusted Fine and Gray model, the relative risk of major haemorrhage was similar among people with HF and AF (HR 1.71, 95% CI 1.66 to 1.78) and AF only (HR 1.82, 95% CI 1.77 to 1.87) (table 4).

The cumulative probability of a first major haemorrhage was highest at each of the prespecified follow-up times for people with HF and AF, irrespective of whether this was estimated by the CIF or KM failure function (table 5, figure 1). Close to one in five of the people with HF and AF who survived to 10 years had a major haemorrhage (18.2%, 95% CI 16.5 to 19.9) (table 5).

**Table 5:** Cumulative probability of a first major haemorrhage by 3 months, 1 year, 5 years and 10 years in people with atrial fibrillation and/or heart failure, estimated using the cumulative incidence function and Kaplan-Meier failure function

	Analysis	3 months	1 year	2 years	5 years	10 years
Neither heart failure nor atrial fibrillation	Cumulative incidence function	0.10 (0.09–0.11)	0.42 (0.41–0.44)	0.87 (0.85–0.90)	2.22 (2.19–2.26)	4.38 (4.33–4.43)
	Kaplan-Meier failure function	0.10 (0.10–0.11)	0.43 (0.41–0.44)	0.88 (0.86–0.91)	2.30 (2.26–2.35)	4.73 (4.65–4.76)
Heart failure only	Cumulative incidence function	0.43 (0.33–0.55)	1.55 (1.34–1.78)	2.92 (2.62–3.25)	6.30 (5.86–6.79)	9.86 (9.32–10.4)
	Kaplan-Meier failure function	0.43 (0.34–0.56)	1.63 (1.41–1.87)	3.20 (2.88–3.57)	7.86 (7.29–8.50)	15.4 (14.4–16.4)
Atrial fibrillation only	Cumulative incidence function	0.38 (0.32–0.46)	1.45 (1.31–1.61)	2.84 (2.64–3.07)	6.47 (6.15–6.82)	11.2 (10.8–11.7)
	Kaplan-Meier failure function	0.39 (0.32–0.47)	1.49 (1.34–1.66)	3.00 (2.79–3.24)	7.39 (7.02–7.80)	14.6 (13.9–15.3)
Heart failure and atrial fibrillation	Cumulative incidence function	0.63 (0.48–0.73)	2.25 (1.96–2.58)	4.13 (3.62–4.47)	8.22 (7.65–8.93)	12.0 (11.3–12.9)
	Kaplan-Meier failure function	0.64 (0.50–0.83)	2.40 (2.10–2.76)	4.68 (4.23–5.24)	11.0 (10.3–12.1)	18.2 (16.5–19.9)

The reported results are a mean calculated across the yearly landmark analyses from index date up to 15-years years follow-up, weighted by the inverse of the variance for each year’s results.

Two landmark analyses were done as a sensitivity analysis where the presence of HF or AF was determined at landmark date and produced similar results (online supplemental table 4).

### Major haemorrhage in relation to anticoagulation

In postestimation analyses, a prescription for either an oral anticoagulant (HR 1.07, 95% CI 1.03 to 1.11) or antiplatelet (HR 1.05, 95% CI 1.02 to 1.07) were associated with an increased risk of bleeding across the study cohort. People with HF and AF who were prescribed an anticoagulant were at nearly threefold greater risk of a major bleed compared with the wider population not prescribed anticoagulation (HR 2.83, 95% CI 2.71 to 2.95) while the risk for people with AF only prescribed an anticoagulant was also high (HR 2.06, 95% CI 1.98 to 2.14).

In a subgroup analysis limiting to the population with AF who were prescribed an oral anticoagulant, HF remained associated with a significant increased hazard of major haemorrhage in both the unadjusted (HR 2.04, 95% CI 1.93 to 2.15) and full Cox models (HR 1.68, 95% CI 1.59 to 1.78).

The proportion of people with AF who were prescribed an anticoagulant was low, with just 33.8% (n=46 974 of 139 175) of people diagnosed with AF and a CHA_2_DS_2_-VASc score ≥2 prescribed an oral anticoagulant across the study period. Most people were prescribed warfarin (89.9%) rather than a DOAC. The proportion of people prescribed anticoagulation increased over time. For example, among people with AF in the cohort from 2014 onwards, 45.6% were prescribed an oral anticoagulant (n=8303 of 18 193). Across follow-up, 75.7% of people with AF were prescribed an anticoagulant or antiplatelet, reflecting international guidelines recommended either in the early years of the study.^21^ Prescribing rates of anticoagulants and antiplatelets were similar among people with AF, with or without HF.

## Discussion

In this large observational study, we found that the relative risk of major haemorrhage was higher among people with HF and AF, compared with people with AF only, including among people prescribed an oral anticoagulant. However, over 60% of people with HF and AF died during follow-up, compared with a quarter of people with AF only. Accounting for this competing risk of death did not alter the risk of major haemorrhage among people with AF only but led to a significant attenuation in the HR for people with HF and AF, so that the relative risk was similar among people with AF, with or without HF. HF was associated with an increased risk of major haemorrhage across age groups, but a greater risk compared with people with AF was seen in those aged under 75 years. Our results imply that the absolute risk of major haemorrhage is higher among people with HF and AF than those with AF only, but prognosis is important to consider when estimating bleeding risk.

### Comparison to the existing literature

Previous research has reported that HF may be associated with an increased risk of bleeding among people with AF.^22 23^ Similar to our results, an observational study using Veteran’s Affairs data in the USA reported that the incidence of bleeding that required hospitalisation among people with HF and AF was 3.25 per 100 person-years among people prescribed warfarin and 2.35 per 100 person-years among those prescribe apixaban.^24^ A separate observational study in Japan reported the incidence of major gastrointestinal bleeding alone was 3.2% among patients with HF over around 3-year follow-up.^25^ In contrast, a recent observational study involving 28 628 patients with AF enrolled in the GARFIELD-AF registry, with 63.3% prescribed an anticoagulant, reported no association between the presence of HF and bleeding risk over 2-year follow-up (HR 1.07%, 95% CI 0.84 to 1.36).^26^

Reflecting this uncertainty, HF is including in some risk scores for major bleeding in anticoagulated patients with AF, such as QBleed, but not the most commonly used, HAS-BLED or ORBIT.^8 9 27^ HF was considered as a candidate variable in the development of HAS-BLED but was not included in the final model as the p value for bleed risk (0.14) exceeded the prespecified threshold of 0.10.^8^ In the ORBIT cohort, HF was significantly associated with risk of major bleeding with the p value for the χ^2^ test <0.000 but was not one of the five factors most closely associated to bleed risk and so was not included in the final model.^9^ Of note, none of these models was developed using competing risks methods.

We have shown that people with HF and AF are at a greater risk of major haemorrhage compared with people with AF only, irrespective of anticoagulation prescription, though this risk is tempered by the high rate of mortality among people with HF. Previous studies that included smaller numbers of participants or had relatively short-term follow-up may have underestimated this association, particularly because the incidence of major haemorrhage is small in comparison to other outcomes, such as all-cause mortality.

### Strengths and limitations

Our study reports the real-world incidence of major haemorrhage among a large contemporary primary care cohort, generalisable to other high-income populations. To our knowledge, this is the first study to account for competing risks in the prediction of bleeding risk among people with AF, despite the relevance of this approach among patients with a poor prognosis, such as those with HF. Previous research among people with AF has highlighted how traditional survival analysis that does not account for competing risks overestimates stroke incidence.^28^

Using routinely collected primary care data relies on the quality of recording in the electronic health record. The diagnosis of HF was very infrequently classified by left ventricular ejection fraction, which precluded an analysis of bleeding risk by HF type. However, previous analysis of bleeding risk between different types of anticoagulant in people with AF and HF has not demonstrated a difference in bleed risk based on left ventricular ejection fraction.^24^ Our definition of major haemorrhage may overestimate the importance of some episodes of bleeding in cases where the bleed was only part of the reason that a patient was admitted to hospital. It was not possible to quantify bleed severity based on the need for transfusion or fall in haemoglobin, as defined in international guidelines. Nonetheless, our codes focus on outcomes that are likely to be relevant to patients and healthcare systems, reflecting hospital admissions and serious bleeding in the community.

Prescription of anticoagulation and antiplatelet treatment is important when estimating bleeding risk and the risk prediction scores for major haemorrhage in people with AF were developed to use in those prescribed an anticoagulant. The proportion of people with AF prescribed an oral anticoagulant was significantly lower than anticipated and relatively few people were prescribed a DOAC. In part, this reflects changes in guidelines and practice over the study period. However, we may also have missed some prescriptions outside our prespecified time windows for data collection (90 days), leading to an underestimate of the effect of these medications on bleeding risk. However, the proportion of people prescribed an anticoagulant was similar among those with AF, with or without HF and adjusting for anticoagulation did not change the association between HF and bleeding risk.

Our analysis assumes that patients who are prescribed anticoagulation continue with this treatment allocation over the course of the study, but it is likely that some patients who were not initially prescribed an anticoagulant would have started it at a later date or that some patients would have stopped taking anticoagulation during follow-up, for example, due to side effects. This applies for other time-varying covariates, such as smoking status or blood pressure. Although we adjusted the Cox and Fine and Gray models for time-varying covariates, this could potentially introduce bias as these covariates may be affected by previous exposure status and treatment.^29^ Marginal structural models can correctly control for time-dependent confounders and so could have been used to analyse the importance of changes in treatment over time, but this was not the focus of this analysis and would be a significant piece of work in its own right.

### Implications of this research

Based on the CHA2DS2VASc stroke risk score, all patients with HF and AF should be considered for anticoagulation.^3 4^ When anticoagulation is initiated, international guidelines recommend that clinicians also undertake a bleeding risk assessment to identify patients at high-risk then seek to address any modifiable bleeding risk factors. In our subgroup analysis of patients with AF who received anticoagulation, those who also had HF were at significantly increased risk of bleeding (HR 1.68, 95% CI 159 to 1.78). However, at present, the most widely recommended bleed risk scores, HAS-BLED and ORBIT, do not include HF.^8 9^ Bleeding risk scores can only help to identify patients with most to gain from risk factor modification if they are accurate. Our results imply bleeding risk may be under-estimated in people with HF and AF using current risk scores.

Our results also show that competing risks are important to consider in determining bleeding risk among people with HF. Overall, the prognosis for people with HF is poor but outlook varies considerably based on an individual’s age.^10^ Among older people who have a poor prognosis, there may only be a small difference in bleed risk between those with HF plus AF compared with people with AF only. However, younger people with HF and AF who have a good prognosis may be at a comparatively higher bleed risk. We hope to undertake further research to update bleeding risk prediction scores, incorporating competing risk methods to take account of mortality over time.

## Conclusion

People with HF and AF are at greater risk of major haemorrhage compared with people with AF only. This is true across age groups and for both gastrointestinal and intracranial haemorrhage types. However, among our cohort, the incidence of all-cause mortality was higher than the incidence of major haemorrhage. Accounting for the competing risk of death led to a greater attenuation in the risk of major haemorrhage among people with HF and AF compared with people with AF only. Our findings suggest that bleed risk scores for patients with AF may need updating to recognise the importance of HF, but development and validation of these models should incorporate competing risk methods.

## supplementary material

10.1136/openhrt-2024-002975 online supplemental table 1

## Data Availability

Data may be obtained from a third party and are not publicly available.
