# Supplementary material for: Heart failure and major haemorrhage in people with atrial fibrillation
Source: Open Heart. 2024 Oct 14;11(2):e002975. doi: 10.1136/openhrt-2024-002975 (PMC11474723; doi:10.1136/openhrt-2024-002975)
Supplement: online supplemental table 1 [file openhrt-11-2-s001.docx]

**Supplemental table 1:** Crude incidence rates of first major haemorrhage per 1,000 person years at risk, by type of haemorrhage and presence of heart failure and/or atrial fibrillation

|  | | | **Crude incidence rates per 1,000 person years at risk** | | | |
| --- | --- | --- | --- | --- | --- | --- |
|  | **Number of participants at risk** | **Number of participants with any bleed** | **First major haemorrhage of any type** | **Intracranial haemorrhage** | **Gastrointestinal haemorrhage** | **Other major haemorrhage** |
| **Neither Heart failure nor atrial fibrillation** | 2,105,773 | 66,410 | 3.53 (3.50-3.56) | 0.66 (0.65-0.67) | 2.53 (2.51-2.56) | 0.28 (0.27-0.29) |
| **Heart failure only** | 79,461 | 5,900 | 19.5 (18.9-20.1) | 2.66 (2.47-2.87) | 15.1 (14.6-15.6) | 1.94 (1.77-2.12) |
| **Atrial fibrillation only** | 126,251 | 10,121 | 17.5 (17.2-17.9) | 4.50 (4.31 to 4.69) | 11.4 (11.1 to 11.7) | 1.80 (1.69 to 1.93) |
| **Heart failure and atrial fibrillation** | 60,270 | 4,995 | 30.7 (29.9-31.6) | 5.80 (5.45-6.17) | 22.5 (21.8-23.2) | 3.79 (3.51-4.09) |

**Supplemental table 2:** Cox regression model for any bleed risk by heart failure and atrial fibrillation

|  | Number of participants at risk | Number of participants with any bleed | Unadjusted | Model 1 | Model 2 | Model 3 |
| --- | --- | --- | --- | --- | --- | --- |
| **Neither Heart failure nor atrial fibrillation** | 2,105,773 | 192,544 | 1 (ref) | 1 (ref) | 1 (ref) | 1 (ref) |
| **Heart failure only** | 74,860 | 8,766 | 3.01 (2.94 – 3.07) | 1.86 (1.81 – 1.90) | 1.63 (1.60 – 1.67) | 1.60 (1.56 – 1.63) |
| **Atrial fibrillation only** | 118,099 | 17,068 | 3.04 (2.99 – 3.09) | 1.94 (1.91 – 1.97) | 1.75 (1.72 – 1.78) | 1.67 (1.64 – 1.70) |
| **Heart failure and atrial fibrillation** | 54,926 | 8,480 | 4.47 (4.37 – 4.56) | 2.52 (2.46 – 2.57) | 2.19 (2.14 – 2.24) | 2.11 (2.06 – 2.16) |

Model 1 – age and sex adjusted.

Model 2 – adjusted for age, sex, hypertension, diabetes, history of thromboembolic or vascular disease, smoking and ethnicity

Model 3 – adjusted for age, sex, hypertension, chronic kidney disease, liver disease, thrombosis, NSAIDs or antiplatelets, alcohol use

**Supplemental table 3:** Sensitivity analysis showing the hazard ratios of first major haemorrhage for people with heart failure and/or atrial fibrillation, comparing a Cox proportional hazards and a Fine and Gray competing risk models. Fatal major haemorrhage events recorded in the Office of National Statistics with no major haemorrhage event coded in CPRD or HES are included as an outcome in this analysis.

|  | **Unadjusted** | | **Model 1** | | **Model 2** | | **Model 3** | |
| --- | --- | --- | --- | --- | --- | --- | --- | --- |
|  | Cox model | Fine & Gray model | Cox model | Fine & Gray model | Cox model | Fine & Gray model | Cox model | Fine & Gray model |
| **Neither heart failure nor atrial fibrillation** | 1 (reference) | | 1 (reference) | | 1 (reference) | | 1 (reference) | |
| **Heart failure only** | 5.40 (5.25 to 5.56) | 3.25 (3.16 to 3.51) | 2.35 (2.27 to 2.42) | 1.69 (1.63 to 1.74) | 2.09 (2.02 to 2.16) | 1.51 (1.46 to 1.56) | 2.06 (1.99 to 2.12) | 1.47 (1.43 to 1.52) |
| **Atrial fibrillation only** | 4.68 (4.57 to 4.79) | 3.83 (3.74 to 3.95) | 2.15 (2.10 to 2.21) | 2.14 (2.09 to 2.20) | 1.96 (1.91 to 2.01) | 1.90 (1.85 to 1.95) | 1.89 (1.84 to 1.94) | 1.83 (1.78 to 1.88) |
| **Heart failure and atrial fibrillation** | 8.28 (8.05 to 8.52) | 4.43 (4.30 to 4.56) | 3.09 (3.00 to 3.19) | 2.09 (2.02 to 2.15) | 2.70 (2.62 to 2.79) | 1.80 (1.74 to 1.86) | 2.63 (2.55 to 2.72) | 1.72 (1.67 to 1.78) |

* Cox model reports the hazard ratio for first major haemorrhage with 95% confidence interval

** Fine & Gray model reports the sub-distribution hazard ratio for first major haemorrhage with 95% confidence interval

Model 1 adjusted for age and sex

Model 2 adjusted for age, sex, hypertension, diabetes, thrombo-embolism or stroke, prior vascular disease including myocardial infarct, ischaemic heart disease, aortic plaque or peripheral arterial disease, smoking and ethnicity.

Model 3 adjusted for age, sex, hypertension, stroke, chronic kidney disease, liver disease, anaemia, labile INR, alcohol use, and prescription of non-steroidal anti-inflammatories or antiplatelet drugs

**Supplemental table 4:** Landmark analysis showing the hazard ratios of first major haemorrhage for people with heart failure and/or atrial fibrillation prior to entry to the study or at 5-year follow-up, comparing a Cox proportional hazards and a Fine and Gray competing risk models.

|  | **Unadjusted** | | **Model 1** | | **Model 2** | | **Model 3** | |
| --- | --- | --- | --- | --- | --- | --- | --- | --- |
|  | Cox model* | Fine & Gray model** | Cox model* | Fine & Gray model** | Cox model* | Fine & Gray model** | Cox model* | Fine & Gray model** |
| **Neither Heart failure nor atrial fibrillation** | 1 (reference) | | 1 (reference) | | 1 (reference) | |  | |
| **Landmark analysis based on a diagnosis of heart failure or atrial fibrillation prior to study index date** | | | | | | | | |
| **Heart failure only** | 4.40 (4.21 to 4.61) | 2.40 (2.29 to 2.51) | 1.76 (1.68 to 1.84) | 1.13 (1.08 to 1.19) | 1.55 (1.48 to 1.63) | 1.04 (1.00 to 1.10) | 1.49 (1.41 to 1.58) | 1.28 (1.21 to 1.36) |
| **Atrial fibrillation only** | 3.77 (3.63 to 3.91) | 2.74 (2.64 to 2.85) | 1.78 (1.71 to 1.85) | 1.45 (1.39 to 1.51) | 1.63 (1.57 to 1.70) | 1.33 (1.28 to 1.39) | 1.58 (1.51 to 1.66) | 1.47 (1.40 to 1.54) |
| **Heart failure and atrial fibrillation** | 6.17 (5.81 to 6.55) | 2.87 (2.70 to 3.05) | 2.33 (2.20 to 2.48) | 1.29 (1.21 to 1.37) | 2.07 (1.95 to 2.20) | 1.17 (1.10 to 1.25) | 2.04 (1.89 to 2.21) | 1.73 (1.59 to 1.88) |
| **Landmark analysis based on a diagnosis of heart failure or atrial fibrillation prior to 5-year follow-up** | | | | | | | | |
| **Heart failure only** | 4.64 (4.43 to 4.86) | 3.24 (3.10 to 3.40) | 1.98 (1.88 to 2.07) | 1.54 (1.47 to 1.62) | 1.69 (1.61 to 1.77) | 1.35 (1.28 to 1.42) | 1.78 (1.68 to 1.89) | 1.37 (1.30 to 1.44) |
| **Atrial fibrillation only** | 3.98 (3.84 to 4.12) | 3.29 (3.17 to 3.41) | 1.85 (1.79 to 1.92) | 1.69 (1.63 to 1.76) | 1.68 (1.62 to 1.74) | 1.54 (1.48 to 1.60) | 1.65 (1.57 to 1.72) | 1.51 (1.46 to 1.57) |
| **Heart failure and atrial fibrillation** | 6.84 (6.49 to 7.21) | 4.17 (3.96 to 4.40) | 2.65 (2.51 to 2.80) | 1.84 (1.74 to 1.95) | 2.29 (2.17 to 2.42) | 1.62 (1.53 to 1.72) | 2.41 (2.25 to 2.58) | 1.67 (1.57 to 1.76) |

* Cox model reports the hazard ratio for first major haemorrhage with 95% confidence interval

** Fine & Gray model reports the sub-distribution hazard ratio for first major haemorrhage with 95% confidence interval

Model 1 adjusted for age and sex

Model 2 adjusted for age, sex, hypertension, diabetes, thrombo-embolism or stroke, prior vascular disease including myocardial infarct, ischaemic heart disease, aortic plaque or peripheral arterial disease, smoking and ethnicity.

Model 3 adjusted for age, sex, hypertension, stroke, chronic kidney disease, liver disease, anaemia, labile INR, alcohol use, and prescription of non-steroidal anti-inflammatories or antiplatelet drugs

**Supplemental table 5:** Incidence rates of first major haemorrhage per 1,000 person years at risk for people with heart failure and/or atrial fibrillation, based on age categories

|  | **Number of patients at risk** | **Number of patients with an first major haemorrhage** | **Incidence rate per 1,000 person years at-risk (95% confidence interval)** |
| --- | --- | --- | --- |
| **Neither Heart failure nor atrial fibrillation** | | | |
| **All ages** |  |  | 3.53 (3.50-3.56) |
| **Age under 65 years** | 1,600,595 | 29,529 | 2.45 (2.43 to 2.48) |
| **Age 65 to 75 years** | 289,892 | 12,999 | 5.93 (5.82 to 6.03) |
| **Age 75 years or older** | 223,499 | 11,708 | 10.4 (10.3 to 10.6) |
| **Heart failure only** | | | |
| **All ages** |  |  | 19.5 (18.9-20.1) |
| **Age under 65 years** | 14,533 | 716 | 11.6 (10.8 to 12.5) |
| **Age 65 to 75 years** | 18,486 | 1,119 | 16.5 (15.5 to 17.5) |
| **Age 75 years or older** | 47,110 | 2,874 | 25.7 (24.8 to 26.7) |
| **Atrial fibrillation only** | | | |
| **All ages** |  |  | 17.5 (17.2-17.9) |
| **Age under 65 years** | 25,743 | 1,185 | 8.82 (8.33 to 9.33) |
| **Age 65 to 75 years** | 33,583 | 2,213 | 14.9 (14.4 to 15.5) |
| **Age 75 years or older** | 68,222 | 4,858 | 26.1 (25.4 to 26.8) |
| **Heart failure and atrial fibrillation** | | | |
| **All ages** |  |  | 30.7 (30.0-31.6) |
| **Age under 65 years** | 5,753 | 432 | 17.3 (15.8 to 19.0) |
| **Age 65 to 75 years** | 11,865 | 1,044 | 23.9 (22.5 to 25.4) |
| **Age 75 years or older** | 43,141 | 3,519 | 37.7 (36.44 to 38.9) |

**Appendix 1.** Exposure and outcome code list for analysis of major haemorrhage in people with heart failure and atrial fibrillation

| **Atrial fibrillation (CPRD)** | | |
| --- | --- | --- |
| Description | medcode | READcode |
| Paroxysmal atrial fibrillation | 1268 | G573200 |
| Permanent atrial fibrillation | 96277 | G573400 |
| Non-rheumatic atrial fibrillation | 35127 | G573300 |
| Atrial fibrillation | 1664 | G573000 |
| Persistent atrial fibrillation | 96076 | G573500 |
| Atrial fibrillation and flutter | 2212 | G573.00 |
| Atrial fibrillation and flutter NOS | 23437 | G573z00 |
| ECG: atrial fibrillation | 3757 | 3272 |
| Paroxysmal atrial fibrillation | 1268 | G573200 |
| Permanent atrial fibrillation | 96277 | G573400 |
| Non-rheumatic atrial fibrillation | 35127 | G573300 |
| Atrial fibrillation | 1664 | G573000 |
| Persistent atrial fibrillation | 96076 | G573500 |
| Atrial fibrillation and flutter | 2212 | G573.00 |
| Atrial fibrillation and flutter NOS | 23437 | G573z00 |
|  |  |  |
| **Heart failure (CPRD)** | | |
| readterm | medcode | readcode |
| H/O: heart failure | 15058 | 14A6.00 |
| H/O: Heart failure in last year | 46912 | 14AM.00 |
| Heart failure confirmed | 9913 | 1O1..00 |
| On optimal heart failure therapy | 111428 | 2JZ..00 |
| New York Heart Assoc classification heart failure symptoms | 46672 | 388D.00 |
| Heart failure self-management plan agreed | 106198 | 661M500 |
| Heart failure 6 month review | 83502 | 662p.00 |
| Congestive heart failure monitoring | 12366 | 662T.00 |
| Heart failure annual review | 30779 | 662W.00 |
| Education about deteriorating heart failure | 105002 | 679W100 |
| Preferred place of care for next exacerbation heart failure | 105542 | 8CeC.00 |
| Heart failure care plan discussed with patient | 32945 | 8CL3.00 |
| Has heart failure management plan | 103732 | 8CMK.00 |
| Heart failure clinical pathway | 106008 | 8CMW800 |
| Admit heart failure emergency | 32898 | 8H2S.00 |
| Heart failure follow-up | 17851 | 8HBE.00 |
| Discharge from practice nurse heart failure clinic | 91288 | 8Hg8.00 |
| Discharge from heart failure nurse service | 102585 | 8HgD.00 |
| Referral to heart failure nurse | 26115 | 8HHb.00 |
| Referral to heart failure exercise programme | 70619 | 8HHz.00 |
| Referred to heart failure education group | 71235 | 8Hk0.00 |
| Referral to heart failure clinic | 48897 | 8HTL.00 |
| Referral to rapid access heart failure clinic | 106680 | 8HTL000 |
| Heart failure rehabilitation programme not available | 110101 | 8I98.00 |
| Referral to heart failure exercise programme not indicated | 106836 | 8IB8.00 |
| Referral to heart failure education group declined | 107981 | 8IE0.00 |
| Referral to heart failure exercise programme declined | 106894 | 8IE1.00 |
| Seen in heart failure clinic | 12627 | 9N0k.00 |
| Seen by community heart failure nurse | 19002 | 9N2p.00 |
| Did not attend practice nurse heart failure clinic | 95021 | 9N4s.00 |
| Did not attend heart failure clinic | 83481 | 9N4w.00 |
| Referred by heart failure nurse specialist | 69062 | 9N6T.00 |
| Heart failure monitoring administration | 32911 | 9Or..00 |
| Heart failure review completed | 19380 | 9Or0.00 |
| Heart failure monitoring telephone invite | 90193 | 9Or1.00 |
| Heart failure monitoring verbal invite | 90192 | 9Or2.00 |
| Heart failure monitoring first letter | 72965 | 9Or3.00 |
| Heart failure monitoring second letter | 72386 | 9Or4.00 |
| Heart failure monitoring third letter | 89650 | 9Or5.00 |
| Hypertensive heart&renal dis wth (congestive) heart failure | 21837 | G232.00 |
| Heart failure | 2062 | G58..00 |
| Cardiac failure | 1223 | G58..11 |
| Congestive heart failure | 398 | G580.00 |
| Congestive cardiac failure | 2906 | G580.11 |
| Right heart failure | 10079 | G580.12 |
| Right ventricular failure | 10154 | G580.13 |
| Biventricular failure | 9524 | G580.14 |
| Chronic congestive heart failure | 32671 | G580100 |
| Decompensated cardiac failure | 27884 | G580200 |
| Compensated cardiac failure | 11424 | G580300 |
| Congestive heart failure due to valvular disease | 94870 | G580400 |
| Left ventricular failure | 884 | G581.00 |
| Heart failure with normal ejection fraction | 101138 | G583.00 |
| HFNEF - heart failure with normal ejection fraction | 101137 | G583.11 |
| Heart failure with preserved ejection fraction | 106897 | G583.12 |
| Heart failure NOS | 4024 | G58z.00 |
| Cardiac failure NOS | 17278 | G58z.12 |
| Post cardiac operation heart failure NOS | 96799 | G5y4z00 |
| Heart failure as a complication of care | 66306 | SP11111 |
| New York Heart Assoc classification heart failure symptoms | 26242 | ZRad.00 |
| Cardiac failure therapy | 24503 | 8B29.00 |
| Excepted heart failure quality indicators: Patient unsuitabl | 30749 | 9hH0.00 |
| Exception reporting: heart failure quality indicators | 90935 | 9hH..00 |
| Heart failure resolved | 100784 | 2126400 |
| Heart failure information given to patient | 60099 | 67D4.00 |
| Excepted heart failure quality indicators: Informed dissent | 64062 | 9hH1.00 |
| Suspected heart failure | 21235 | 1J60.00 |
| Acute congestive heart failure | 23707 | G580000 |
| Acute heart failure | 27964 | G582.00 |
| Cardiac failure following abortive pregnancy | 112120 | L09y200 |
| Right ventricular failure | 104275 | G584.00 |
| Impaired left ventricular function | 5942 | G581.13 |
| Acute left ventricular failure | 5255 | G581000 |
| New York Heart Association classification - class II | 13189 | 662g.00 |
| New York Heart Association classification - class I | 18853 | 662f.00 |
| New York Heart Association classification - class IV | 51214 | 662i.00 |
| New York Heart Association classification - class III | 19066 | 662h.00 |
|  |  |  |
| **Intracranial haemorrhage (CPRD)** | | |
| readterm | medcode | readcode |
| Intracerebral haemorrhage | 5051 | G61..00 |
| CVA - cerebrovascular accid due to intracerebral haemorrhage | 6960 | G61..11 |
| Stroke due to intracerebral haemorrhage | 18604 | G61..12 |
| Intracerebral haemorrhage NOS | 3535 | G61z.00 |
| Cerebellar haemorrhage | 13564 | G613.00 |
| Intracranial haemorrhage NOS | 20284 | G62z.00 |
| "Intracerebral haemorrhage, intraventricular" | 30202 | G617.00 |
| Cerebral haemorrhage following injury | 5682 | S62..00 |
| Perinatal intraventricular haemorrhage | 25415 | Q411.00 |
| Intra-ocular haemorrhage | 2629 | F404500 |
| Subarachnoid haemorrhage from middle cerebral artery | 19412 | G602.00 |
| Evacuation of intracerebral haematoma NEC | 7017 | 7004300 |
| "Subarachnoid haemorrh from intracranial artery, unspecif" | 17326 | G60X.00 |
| "Left sided intracerebral haemorrhage, unspecified" | 28314 | G61X000 |
| "Right sided intracerebral haemorrhage, unspecified" | 19201 | G61X100 |
| Haemorrhagic stroke monitoring | 28914 | 662o.00 |
| Other and unspecified intracranial haemorrhage | 31805 | G62..00 |
| Sequelae of other nontraumatic intracranial haemorrhage | 43451 | G682.00 |
| "Intracerebral haemorrhage in hemisphere, unspecified" | 31060 | G61X.00 |
| Intracranial haemorrhage in fetus or newborn | 43005 | Q200012 |
| "Intrapartum haemorrhage, unspecified" | 32339 | L3X..00 |
| "Cerebral haemorrhage unspecified, due to birth trauma" | 23447 | Q200000 |
| Lobar cerebral haemorrhage | 107440 | G619.00 |
| Traumatic cerebral haemorrhage | 28077 | S62..14 |
| Cerebral haemorrhage following injury NOS | 46545 | S62z.00 |
| Subdural and cerebral haemorrhage due to birth trauma | 34694 | Q200.00 |
| Sequelae of intracerebral haemorrhage | 48149 | G681.00 |
| "Intracerebral haemorrhage, multiple localized" | 57315 | G618.00 |
| [X]Other intracerebral haemorrhage | 53810 | Gyu6200 |
| Intracerebral haemorrhage in fetus or newborn | 36559 | Q200011 |
| Intracranial nontraumatic haemorrhage of fetus and newborn | 35777 | Q417.00 |
| Intraventricular haemorrhage due to birth injury | 57771 | Q411300 |
| Intra-operative haemorrhage | 28652 | SP21000 |
| Subdural or cerebral haemorrhage due to birth trauma NOS | 67184 | Q200z00 |
| Intracerebral (nontraumatic) haemorrhage of fet and newborn | 57783 | Q417000 |
| "[X]Intracerebral haemorrhage in hemisphere, unspecified" | 96630 | Gyu6F00 |
| Other cerebral haemorrhage following injury | 52968 | S63..00 |
| Cerebral haemorrhage due to birth injury | 48731 | Q200700 |
| Cerebellar (nontraum) and post fossa haemorhage fet newborn | 19577 | Q417100 |
| Subdural or cerebral haemorrhage due to birth trauma OS | 63806 | Q200y00 |
| Other cerebral haemorrhage following injury NOS | 42283 | S63z.00 |
| Intrapartum haemorrhage with coagulation defect | 62121 | L3A..00 |
| Massive epicranial subaponeurotic haemorrhage-birth trauma | 104636 | Q201300 |
| [X]Subarachnoid haemorrhage from other intracranial arteries | 108668 | Gyu6000 |
| "[X]Subarachnoid haemorrh from intracranial artery, unspecif" | 108630 | Gyu6E00 |
| [X]Oth intracranial(nontraumatic)haemorrhages/fetus+newborn | 66571 | Qyu5200 |
| [X]Intracranial nontraumatic haemorrhage fetus newborn unsp | 85238 | Qyu5F00 |
| [X]Oth intracranial laceratns+haemorrhages due/birth injury | 112248 | Qyu2000 |
| Basal nucleus haemorrhage | 46316 | G612.00 |
| Bulbar haemorrhage | 62342 | G615.00 |
| Cortical haemorrhage | 31595 | G610.00 |
| External capsule haemorrhage | 30045 | G616.00 |
| Internal capsule haemorrhage | 40338 | G611.00 |
| Pontine haemorrhage | 7912 | G614.00 |
| Subarachnoid haemorrhage | 1786 | G60..00 |
| Ruptured berry aneurysm | 29939 | G600.00 |
| Subarachnoid haemorrhage from carotid siphon and bifurcation | 56007 | G601.00 |
| Subarachnoid haemorrhage from anterior communicating artery | 42331 | G603.00 |
| Subarachnoid haemorrhage from basilar artery | 41910 | G605.00 |
| Subarachnoid haemorrhage NOS | 23580 | G60z.00 |
| Subarachnoid haemorrhage from vertebral artery | 60692 | G606.00 |
| Subarachnoid haemorrhage from posterior communicating artery | 9696 | G604.00 |
| Sequelae of subarachnoid haemorrhage | 44740 | G680.00 |
|  |  |  |
| **Major gastrointestinal haemorrhage (CPRD)** | | |
| readterm | medcode | readcode |
| Oesophageal varices with bleeding | 24989 | G850.00 |
| Oesophageal varices with bleeding in diseases EC | 96756 | G852000 |
| GIB - Gastrointestinal bleeding | 1642 | J68z.11 |
| Gastrotomy and ligation of bleeding point of stomach | 23813 | 7619100 |
| Bleeding chronic duodenal ulcer | 18625 | J121111 |
| Haemorrhage of oesophagus | 16114 | J10y000 |
| Gastrointestinal haemorrhage | 3097 | J68..00 |
| Perinatal gastrointestinal haemorrhage | 71590 | Q414.00 |
| Intestinal haemorrhage NOS | 2150 | J68z100 |
| Gastrointestinal haemorrhage unspecified | 12471 | J68z.00 |
| Gastrointestinal tract haemorrhage NOS | 4636 | J68zz00 |
| Upper gastrointestinal haemorrhage | 4354 | J68z200 |
| Acute gastrojejunal ulcer with haemorrhage | 96628 | J140100 |
| Unspecified gastrojejunal ulcer with haemorrhage | 60346 | J14y100 |
| Acute gastrojejunal ulcer with haemorrhage and perforation | 106330 | J140300 |
| Chronic gastrojejunal ulcer with haemorrhage and perforation | 110244 | J141300 |
| Chronic duodenal ulcer with haemorrhage and perforation | 71881 | J121300 |
| Unspec duodenal ulcer; unspec haemorrhage and/or perforation | 28366 | J12yy00 |
| Unspecified duodenal ulcer with haemorrhage | 2814 | J12y100 |
| Chronic duodenal ulcer with haemorrhage | 48951 | J121100 |
| Unspecified duodenal ulcer with haemorrhage and perforation | 93436 | J12y300 |
| Acute duodenal ulcer with haemorrhage and perforation | 48730 | J120300 |
| Acute duodenal ulcer with haemorrhage | 18001 | J120100 |
| Gastric haemorrhage NOS | 15517 | J68z000 |
| Melaena | 397 | J681.00 |
| Haematemesis | 1188 | J680.00 |
| Vomiting of blood | 2712 | J680.11 |
| Chronic gastric ulcer with haemorrhage and perforation | 71897 | J111300 |
| Bleeding chronic gastric ulcer | 36583 | J111111 |
| Acute gastric ulcer with haemorrhage | 30054 | J110100 |
| Chronic gastric ulcer with haemorrhage | 63582 | J111100 |
| Perforated chronic gastric ulcer | 11104 | J111211 |
| Bleeding acute gastric ulcer | 11124 | J110111 |
| Endoscopic injection haemostasis of gastric ulcer | 63718 | 761D600 |
| Acute gastric ulcer with haemorrhage and perforation | 71403 | J110300 |
| Unspecified gastric ulcer with haemorrhage | 57958 | J11y100 |
| Unspec gastric ulcer; unspec haemorrhage and/or perforation | 94397 | J11yy00 |
| Unspecified peptic ulcer with haemorrhage and perforation | 96622 | J13y300 |
| Acute peptic ulcer with haemorrhage and perforation | 45304 | J130300 |
| Endoscopic injection haemostasis of duodenal ulcer | 45981 | 761D500 |
| Chronic peptic ulcer with haemorrhage | 53126 | J131100 |
| Acute peptic ulcer with haemorrhage | 44637 | J130100 |
| Unspecified peptic ulcer with haemorrhage | 70456 | J13y100 |
| Acute haemorrhagic gastritis | 29492 | J150000 |
| H/O: haematemesis | 5409 | 14C8.00 |
| H/O: upper GIT bleed | 17218 | 14CD.11 |
| H/O: upper GIT haemorrhage | 34466 | 14CD.00 |
| Fibreopt endoscop rubber band ligation of upper GIT varices | 89717 | 761D800 |
| Fibreoptic endoscopic cauterisation lesion of upper GI tract | 18259 | 761D200 |
| Fibreoptic endoscopic sclerotherapy to lesion upper GI tract | 46890 | 761D300 |
| C/O - melaena | 18313 | 19E4.12 |
| H/O: melaena | 375 | 14C9.00 |
| Melaena - O/E of faeces | 37299 | 4737.11 |
| Gastric angiodysplasia | 12568 | J17y900 |
| Angiodysplasia of colon | 6504 | J577000 |
| Bleeding diverticulosis | 2044 | J510900 |
|  |  |  |
| **Other major haemorrhage (CPRD)** | | |
| readterm | medcode | readcode |
| Perinatal haemoptysis | 55759 | Q313300 |
| Massive haemoptysis | 107548 | 1720.00 |
|  |  |  |
| **Intracranial haemorrhage ICD-9 codes (HES)** | | |
| Subarachnoid | 430 |  |
| Intracerebral | 431 |  |
| Other and unspecified intracranial bleeding | 432.0 |  |
| Subdural | 432.1 |  |
| Unspecified intracranial bleeding | 432.9 |  |
|  |  |  |
| **Intracranial haemorrhage ICD-10 codes (HES)** | | |
| Subarachnoid | I60 |  |
| Intracerebral | I61 |  |
| Subdural | I62.0 |  |
| Non-traumatic extradural | I62.1 |  |
| Intracranial, nontraumatic, unspecified | I62.9 |  |
|  |  |  |
| **Gastrointestinal major haemorrhage ICD-9 codes (HES)** | | |
| Acute gastric ulcer with haemorrhage | 531.0 |  |
| Acute gastric ulcer with haemorrhage and perforation | 531.2 |  |
| Chronic or unspecified gastric ulcer with haemorrhage | 531.4 |  |
| Chronic or unspecified gastric ulcer with haemorrhage and perforation | 531.6 |  |
| Acute duodenal ulcer with haemorrhage | 532.0 |  |
| Acute duodenal ulcer with haemorrhage and perforation | 532.2 |  |
| Chronic or unspecified duodenal ulcer with haemorrhage | 532.4 |  |
| Chronic or unspecified duodenal ulcer with haemorrhage and perforation | 532.6 |  |
| Acute peptic ulcer of unspecified site with haemorrhage | 533.0 |  |
| Acute peptic ulcer of unspecified site with haemorrhage and perforation | 533.2 |  |
| Chronic or unspecified peptic ulcer of unspecified site with haemorrhage | 533.4 |  |
| Chronic or unspecified peptic ulcer of unspecified site with haemorrhage and perforation | 533.6 |  |
| Acute gastrojejunal ulcer with haemorrhage | 534.0 |  |
| Acute gastrojejunal ulcer with haemorrhage and perforation | 534.2 |  |
| Chronic or unspecified gastrojejunal ulcer with haemorrhage | 534.4 |  |
| Chronic or unspecified gastrojejunal ulcer with haemorrhage and perforation | 534.6 |  |
| Hematemesis | 578.0 |  |
| Bleeding of gastrointestinal tract unspecified | 578.9 |  |
|  |  |  |
| **Gastrointestinal major haemorrhage ICD-10 codes (HES)** | | |
| Haematemesis | K92.0 |  |
| Melaena | K92.1 |  |
| Oesophageal varices with bleeding | I85.0 |  |
| Oesophageal varices in diseases classified elsewhere with bleeding | I98.20 |  |
| Oesophageal varices with bleeding in disease classified elsewhere | I98.3 |  |
| Ulcer of oesophagus, acute with bleeding | K22.10 |  |
| Ulcer of oesophagus, acute with both bleeding and perforation | K22.12 |  |
| Ulcer of oesophagus, chronic or unspecified with bleeding | K22.14 |  |
| Ulcer of oesophagus, chronic or unspecified with both bleeding and perforation | K22.16 |  |
| Gastric ulcer, acute with bleeding | K25.0 |  |
| Gastric ulcer, acute with both bleeding and perforation | K25.2 |  |
| Gastric ulcer, chronic or unspecified with bleeding | K25.4 |  |
| Gastric ulcer, chronic or unspecified with both bleeding and perforation | K25.6 |  |
| Duodenal ulcer, acute with bleeding | K26.0 |  |
| Duodenal ulcer, acute with both bleeding and perforation | K26.2 |  |
| Duodenal ulcer, chronic or unspecified with bleeding | K26.4 |  |
| Duodenal ulcer, chronic or unspecified with both bleeding and perforation | K26.6 |  |
| Peptic ulcer, acute with bleeding | K27.0 |  |
| Peptic ulcer, acute with both bleeding and perforation | K27.2 |  |
| Peptic ulcer, chronic or unspecified with bleeding | K27.4 |  |
| Peptic ulcer, chronic or unspecified with both bleeding and perforation | K27.6 |  |
| Gastrojejunal ulcer, acute with bleeding | K28.0 |  |
| Gastrojejunal ulcer, acute with both bleeding and perforation | K28.2 |  |
| Gastrojejunal ulcer, chronic or unspecified with bleeding | K28.4 |  |
| Gastrojejunal ulcer, chronic or unspecified with both bleeding and perforation | K28.6 |  |
| Acute bleeding gastritis | K29.0 |  |
| Angiodysplasia of small intestine, except duodenum with bleeding | K63.80 |  |
| Angiodysplasia of stomach and duodenum with bleeding | K31.80 |  |
| Angiodysplasia of colon with bleeding | K55.20 |  |
| Gastrointestinal bleeding, unspecified | K92.2 |  |
|  |  |  |
| **Other major haemorrhage ICD-9 codes (HES)** | | |
| Hemarthrosis site unspecified | 719.1 |  |
| Hemoptysis, unspecified | 786.3 |  |
| Bleeding from throat | 784.8 |  |
|  |  |  |
| **Other major haemorrhage ICD-10 codes (HES)** | | |
| Haemoperitoneum | K66.1 |  |
| Bleeding from throat | R04.1 |  |
| Haemoptysis | R04.2 |  |
| Bleeding from other sites in respiratory passages | R04.8 |  |
| Bleeding from respiratory passages, unspecified | R04.9 |  |
| Haemorrhagic disorder due to circulating anticoagulants | D68.3 |  |
| Haemarthrosis | M25.0 |  |
|  |  |  |
| **Gastrointestinal non-major haemorrhage (CPRD)** | | |
| readterm | medcode | readcode |
| Haemorrhage of rectum and anus | 19271 | J573.00 |
| Haemorrhage of rectum and anus NOS | 46479 | J573z00 |
| PRB - Rectal bleeding | 6554 | J573012 |
| Painless rectal bleeding | 11698 | 196C.00 |
| Rectal bleeding | 621 | J573011 |
| Referral to rectal bleeding clinic | 104124 | 8HTE000 |
| Painful rectal bleeding | 11718 | 196B.00 |
| Rectal haemorrhage | 6574 | J573000 |
|  |  |  |
| **Other non-major haemorrhage (CPRD)** | | |
| readterm | medcode | readcode |
| Urine blood test = ++ | 19792 | 4696.00 |
| Urine blood test = +++ | 13929 | 4697.00 |
| Recurrent benign haematuria syndrome | 7164 | K032100 |
| Recurrent and persistent haematuria, dense deposit disease | 44541 | K0A2600 |
| Recur+persist haematuria difus crescentic glomerulonephritis | 60856 | K0A2700 |
| Painless haematuria | 9651 | K197000 |
| Referral to haematuria clinic | 107296 | 8T10.00 |
| H/O: haematuria | 6247 | 14D5.00 |
| Recurrent and persistent haematuria | 17060 | K0A2.00 |
| Essential haematuria | 47228 | K197.12 |
| Traumatic haematuria | 19361 | K197.11 |
| Painful haematuria | 20357 | K197100 |
| Benign familial haematuria | 105453 | K197500 |
| Recur+persist haematuria, focal+segmental glomerular lesions | 68364 | K0A2100 |
| Blood in urine - haematuria | 6659 | 1A45.00 |
| Frank haematuria | 7232 | K197300 |
| Haematuria | 507 | K197.00 |
| Clot haematuria | 6901 | K197400 |
| Blood in urine - symptom | 6234 | 1A45.11 |
| Blood in urine test | 13913 | 469..11 |
| Recurrent+persistnt haematuria minor glomerular abnormality | 95546 | K0A2000 |
| Haematuria - symptom | 6030 | 1A45.12 |
| Recur+persist haematuria difus membranous glomerulonephritis | 61317 | K0A2200 |
| Recur+persist haemuria df mesangial prolif glomerulnephritis | 49642 | K0A2300 |
| Recur+persist hmuria df mesangiocapilary glomerulonephritis | 60484 | K0A2500 |
| Blood in sputum - haemoptysis | 4135 | 172..00 |
| [D]Haemoptysis | 2244 | R063.00 |
| [D]Haemoptysis NOS | 33742 | R063z00 |
| Haemoptysis - symptom | 1610 | 172..12 |
| Blood in sputum - symptom | 10013 | 172..11 |
| [D]Cough with haemorrhage | 8239 | R063000 |
| [D]Pulmonary haemorrhage NOS | 7285 | R063100 |
|  |  |  |
| **Other non-major haemorrhage ICD-9 codes (HES)** | | |
| Hematuria, unspecified | 599.7 |  |
| Other unspecified hemorrhagic conditions | 287.8 |  |
| Unspecified hemorrhagic conditions | 287.9 |  |
| Bleeding into bladder wall | 596.7 |  |
| Haemorrhage (non-traumatic) eye | 360.43 |  |
|  |  |  |
| **Other non-major haemorrhage ICD-10 codes (HES)** | | |
| Retinal bleeding | H35.6 |  |
| Vitreous bleeding | H43.1 |  |
| Vitreous bleeding in diseases classified elsewhere | H45.0 |  |
| Other specified abnormal uterine and vaginal bleeding | N93.8 |  |
| Abnormal uterine and vaginal bleeding, unspecified | N93.9 |  |
| Gross hematuria | R31.0 |  |
| Recurrent and persistent haematuria, other | N02.8 |  |
| Recurrent and persistent haematuria, unspecified | N02.9 |  |
|  |  |  |
|  |  |  |
